# Dapagliflozin increases the lean-to total mass ratio in type 2 diabetes mellitus

**DOI:** 10.1038/s41387-021-00160-5

**Published:** 2021-06-12

**Authors:** Vaneza Lira W. Wolf, Ikaro Breder, Luiz Sérgio F. de Carvalho, Alexandre A. S. Soares, Riobaldo M. Cintra, Joaquim Barreto, Daniel B. Munhoz, Sheila T. Kimura-Medorima, Wilson Nadruz, Gil Guerra-Júnior, Thiago Quinaglia, Elza Muscelli, Andrei C. Sposito, on behalf of Addenda-BHS2 trial investigators

**Affiliations:** 1 Atherosclerosis and Vascular Biology Laboratory (Atherolab), Cardiology Department, State University of Campinas (Unicamp), Campinas, SP Brazil; 2 Directory of Clinical Research and Innovation, Institute for Strategic Management in Healthcare (IGESDF), Brasília, DF Brazil; 3 Cardiology Division, University of Brasilia Medical School, Brasilia, DF Brazil; 4 Growth and Body Composition Lab, Center for Investigation in Pediatrics, Faculty of Medical Sciences, University of Campinas, Campinas, São Paulo Brazil

**Keywords:** Randomized controlled trials, Diabetes complications

## Abstract

We compared the effect of dapagliflozin versus glibenclamide on the ratio of lean-to total mass in patients with type 2 diabetes mellitus, carotid subclinical atherosclerosis, HbA1c 7.0–9.0% and 40–70 years-old. Ninety-eight patients (61% male; mean age 57 ± 7 years) were randomized into dapagliflozin 10 mg/day or glibenclamide 5 mg/day on top of metformin. Body composition was measured by Dual Energy X-Ray at randomization and after 12 weeks of treatment. Glycemic control was equivalent in both groups. Dapagliflozin decreased total body mass (−2741 g [95% CI: −3360 to 1945]; *p* < 0.001) and lean mass (−347 g [95% CI: −761 to −106]; *p* < 0.001), while glibenclamide increased total body mass (1060 g [95% CI: 140 to 1836]; *p* < 0.001) and lean mass (929 g [95% CI: 575 to 1283]; *p* < 0.001) for the differences between arms. The lean-to-total mass ratio increased by 1.2% in the dapagliflozin group and 0,018% in the glibenclamide group (*p* < 0.001). Dapagliflozin reduced the risk of a negative balance in the lean-to total mass ratio [OR: 0.16 (95% CI: 0.05 to 0.45); *p* < 0.001] even after adjustment for baseline lean-to total mass ratio, waist circumference, HOMAIR, HbA1c, mean of the two hands handgrip strength and gait speed [OR: 0.13 (95% CI: 0.03–0.57); *p* < 0.007]. In conclusion, under equivalent glycemic control, dapagliflozin reduced total body mass but increased the ratio of lean-to-total mass when compared with glibenclamide.

## Introduction

Inhibitors of sodium/glucose cotransporter 2 (SGLT2i) have captured the attention of clinicians worldwide as they reduce the risk of major adverse cardiovascular events [1]. Clinical trials comparing SGLT2i with placebo also revealed that these drugs reduce body weight during the first weeks of administration, mostly due to the negative energy balance caused by increased glycosuria [1, 2]. In these trials, the decline in lean mass was equally reported, which can be assumed to be an unfavorable event according to the change in proportionality between lean mass and total mass [3, 4].

Individuals with type 2 diabetes mellitus (T2DM) present with progressive decline in muscle mass and function, which is associated with insulin resistance, chronic hyperglycemia, reduced mitochondrial function, and microvascular dysfunction [5]. This process predisposes to sarcopenia and, by this way, increased cardiovascular mortality [6]. Glycemic control, particularly by treatment with insulin, increases muscle glucose disposal and mitigates the development or intensification of sarcopenia [7].

Although some controversy exists [8], the general concept is that to compensate for glucose loss and maintain energy substrates, SGLT2i increases gluconeogenesis, at least in part, by increasing glucagon levels and shifting substrate utilization from carbohydrates to lipids and ketone bodies [9]. In addition, an improvement in muscle insulin sensitivity has been reported after SGLT2i [10]. Whether these metabolic adaptations compensate or not the negative calorie balance on lean body mass, we presently do not know. Hence, the present study is a pre-specified analysis of a randomized clinical trial with T2DM subjects, aimed at verifying the effect of dapagliflozin on lean mass when compared with the equipotent glucose-lowering treatment with glibenclamide, during a period of 12 weeks.

## Methods

This study is a pre-specified analysis of the ADDENDA-BHS2 trial (ClinicalTrials.gov, NCT 02919345). Methods and baseline characteristics have been published elsewhere [11]. Briefly, ADDENDA-BHS2 was a randomized, single-center, open, active-controlled, phase-4 trial that compared dapagliflozin versus glibenclamide for 12 weeks. Inclusion criteria were: (i) high cardiovascular risk, defined by stable coronary artery disease or subclinical carotid atherosclerotic disease; (ii) T2DM taking up to two oral hypoglycemic agents; (iii) HbA1c between 7% and 9% at the time of randomization; and (iv) age between 40 and 70 years. The study protocol was approved by the Institutional Review Board, and all participants provided written informed consent.

Before randomization, antidiabetic and antihypertensive medications were adjusted in a 16-week run-in period. Randomization was performed in a 1:1 ratio for dapagliflozin 10 mg/day or glibenclamide 5 mg/day on top of metformin for 12 weeks of intervention. Eligible volunteers received a 30-day supply of medications according to a previously published protocol [11]. Anthropometric evaluation performed by the investigators was performed within 2 weeks prior to the randomization visit and up to 2 weeks after the last visit.

Blood samples were obtained following a 12-h overnight fasting at randomization and at the 12-week visit and were centrifuged at 3500 rpm for 10 min at 4 °C. Serum aliquots were measured for glucose (Cobas c702 Roche, Germany) and insulin (Cobas e602 Roche, Germany). The Homeostasis Model Assessment for Insulin Sensitivity (HOMA-2S) was calculated using the Levy formula [12]. Glycated hemoglobin (HbA1c) was measured by high-performance liquid chromatography (D-100, Bio-rad, Brazil).

Body composition was assessed by dual energy X-ray absorptiometry (DXA), model iDXA (GE Healthcare Lunar, Madison, WI, USA) with fan-beam detectors. After the evaluation, the data were analyzed using the enCoretm 2011 software, version 13.60 (GE Healthcare Lunar, Madison, WI, USA). The positioning and placement of regions of interest followed the specifications recommended by the manufacturer. The lean mass ratio was calculated by applying the formula: total lean mass/total body mass and peripheral lean mass was calculated by adding the lean mass of the legs to that of the arms [13].

### Statistical analysis

The results of normally distributed continuous variables, as determined by the Shapiro-Wilk test, are expressed as the mean±standard deviation (SD), and those of continuous variables with nonparametric distributions are expressed as median and interquartile range (IQR). Baseline characteristics of the two groups are presented as mean and 95% confidence interval (CI). Changes from baseline were compared between treatments by analysis of covariance (ANCOVA) adjusted for baseline values. Prerequisites for the ANCOVA models (linearity, distribution normality and equal variance) were checked using histograms, normal probability graphs and residual dispersion. Binomial logistic regression was used to investigate predictors for the decline in the lean-to-total mass ratio, a proxy for increased risk of sarcopenia. Logistic regression analyses were also adjusted for the variables that showed a difference in the baseline. A *p*-value <0.05 was considered as statistically significant. Data analysis was performed using the statistical software, R3.6.2.

## Results

Of the 674 subjects screened, 134 were selected in the run-in phase, 98 individuals were enrolled into the trial, and 89 patients completed both pre- and post-randomization DXA evaluations, 45 in the glibenclamide group and 44 in the dapagliflozin group. One patient dropped out during the treatment period and eight patients refused to undergo the DXAs. The baseline characteristics are presented in Table 1. There were no differences at baseline in anthropometric measurements, body composition, and glycemic control across groups.

**Table 1:** Characteristics of the two groups at baseline.

	Dapagliflozin	Glibenclamide
*N*	44	45
Age (years)	58 ± 7	58 ± 7
Male (%)	27 (61)	27 (60)
BMI (kg/m^2^)	30 (7)	31 (7)
Lean mass/total mass ratio	0.6 (0.1)	0.6 (0.1)
Total lean mass (kg)	51.3 ± 9.5	49.2 ± 8.0
Peripheral lean mass (kg)	23.3 ± 4.9	22.2 ± 4.4
Total fat mass (kg)	29.9 (12.9)	28.3 (12.0)
Total body mass (kg)	81.6 (19.9)	83.2 (15.9)
Fasting blood glucose (mg/dL)	165 (66)	171 (70)
Fasting plasma insulin (mUI/L)	13 (10)	14 (10)
HOMA-2S	50 (37)	50 (40)
HbA1c (%)	7.7 (1.2)	7.9 (1.4)
Adiponectin (ng/mL)	11.1 (11.9)	6.4 (10.3)
CRP (mg/dL)	0.2 (0.2)	0.3 (0.4)

The changes and the difference between the changes in the studied parameters are displayed in Table 2. The change in total body weight after 12 weeks of treatment was different between dapagliflozin (−2.7 kg 95% CI: −3.4 to −1.9) and glibenclamide arms (1.1 kg 95% CI: 0.2 to 1.8; *p* < 0.0001). Fat mass change was also different between dapagliflozin group (−2.0 kg [95% CI: −2.3 to 1.9]; *p* < 0.001) and glibenclamide group (0.4 kg [95% CI: 0.1 to 0.7]; *p* < 0.001). Likewise, lean mass change was different between dapagliflozin (−0.3 kg [95% CI: −0.8 to −0.1]; *p* < 0.001) and glibenclamide (0.9 kg [95% CI: 0.6 to 1.3]; *p* < 0.001). The proportion of lean-to-total mass increased by 1.2% in the dapagliflozin arm and increased by 0.02% in the glibenclamide group (between groups difference *p* < 0.001). These differences in the change of total body weight, fat mass, lean mass, and lean-to-total mass between treatments remained significant after adjustment for the baseline values of these variables. Consistently, there were no differences in the change in the lean-to-total mass in the dapagliflozin group for those who had a lean-to-total mass ratio below or above the 50th percentile at randomization (OR: 1.02 [95% CI: 0.00–2.38]; *p* = 0.517).

**Table 2:** Changes in primary and secondary outcomes.

	Dapagliflozin	Glibenclamide	95% CI of the difference	*p*-value
*N*	44	45		
BMI (kg/m^2^)	−1.0 (−1.2 to 0.7)	0.4 (0.1 to 0.7)	−1.39 (−1.76 to −1.01)	<0.001
Lean mass/total mass ratio	0.012 (0.00 to 0.15)	0.001 (−0.00 to 0.00)	0.011 (0.006 to 0.015)	<0.001
Total lean mass (g)	−347 (−761 to −106)	929 (575 to 1283)	−0.41 (−0.60 to −0.23)	<0.001
Peripheral lean mass (g)	−391 (−615 to −166)	450 (188 to 711)	−840 (−1181 to −500)	<0.001
Total Fat mass (g)	−1959 (−2307 to −1429)	413 (76 to 749)	−2372 (−2916 to −1827)	<0.001
Total body mass (g)	−2741 (−3360–1945)	1060 (140–1836)	−3535 (−4339 to −2732)	<0.001
Fasting blood glucose (mg/dL)	−40 (−57 to −31)	−30 (−43 to −14)	−10 (−27 to 7)	0.257
Fasting plasma insulin (mUI/L)	−2.1 (−3.8 to −0.5)	−0.6 (−4.5 to 3.3)	−1.7 (−5.5 to 2.1)	0.003
HOMA-2S (%)	19 (4 to 29)	2 (−11 to 11)	16 (0.5 to 33)	0.004
HbA1c (%)	−0.7 (−1.1 to −0.6)	−0.8 (−1.0 to −0.6)	0.29 (−0.28 to 0.34)	0.741
Adiponectin (ng/mL)	0.8 (−0.9 to 2.0)	−0.6 (−1.8 to −0.62)	2.1 (0.2 to 3.9)	0.027
CRP (mg/dL)	0.2 (−0.2 to 0.6)	−0.1 (−0.2 to −0.1)	0.2 (−0.1 to 0.6)	0.222

*cm* centimeters, *g* grams, *Kg* kilogram, *HOMA-2* *S* Homeostasis Model Assessment for insulin sensitivity, *HbA1c* Glycated hemoglobin, *CRP* C-reactive protein; data are means and 95% confidence interval; p: statistical significance.

Binary logistic regression was used to investigate predictors for the decline in lean/total mass after 12 weeks of treatment, considering together the patients enrolled in the two arms of the study. In this analysis, for each 1 kg in total weight increase after the 12-week period of treatment, the risk of decreasing lean-to total mass ratio increased by 23% (95% CI: 6% to 40%, *p* = 0.014). This association lost its significance when treatment with dapagliflozin or glibenclamide was included in the model. Body weight reduction was not associated with the change in lean-to-total mass ratio (OR: 0.20 [95% CI: 0.010 to 1.12]; *p* = 0.134). Dapagliflozin reduced this risk of decreasing the lean-to-total mass ratio when compared with glibenclamide (OR: 0.16 [95% CI: 0.05 to 0.45]; *p* < 0.001). This association remained significant even after adjustment for baseline handgrip strength, total body weight change, and lean-to-total mass at baseline (OR: 0.14 [95% CI: 0.04 to 0.46]; *p* < 0.001).

## Discussion

The present study indicates that in 12 weeks of treatment the use of dapagliflozin when compared to glibenclamide increases the proportion of lean-to-total mass despite decreasing the total lean mass. This effect results from a proportionally greater reduction in fat mass than in lean mass by dapagliflozin and is present even in individuals with reduced lean mass. Potentially, this finding may be clinically relevant for a large portion of T2DM patients, such as those with long-standing T2DM, the elderlies or individuals with chronic kidney disease or heart failure [6, 7].

Upon the administration of SGLT2 inhibitors, a decline in body weight is expected during the first two to four weeks, followed by a reduction in fat mass and small reductions in lean mass that persist in the long term [2, 14]. This is consistent with a bioimpedance spectroscopy-based study [14], which reported that SGLT2 inhibitors (dapagliflozin and empagliflozin) administration mostly resulted in a decrease in adipose tissue mass in 6 months. In line with our results, an average two-thirds of weight loss is derived from adipose tissue, which corresponds to approximately 2 to 3 Kg^2^.

Several prospective studies [5, 15, 16] have suggested that glycemic control, reduction of insulin resistance, and adiponectin [7] have been associated with reduced risk of muscle loss in T2DM individuals and also with improved muscular function. These effects were due to increased insulin sensitivity, reduction of intramuscular lipids [15], oxidative stress [17], and improvement of peripheral microvascular function [16]. Previous studies compared SGLT2 inhibitors to placebo and therefore could not exclude the potential impact of glycemic control on changes in body composition [4]. In the present study, we sought to avoid this confusing effect by comparing dapagliflozin with glibenclamide under conditions of equivalent glucose-lowering effect. Under these conditions, dapagliflozin increased insulin sensitivity, which may have contributed to the higher proportion of lean-to-total mass, despite the absolute reduction in total and lean mass as compared with glibenclamide therapy.

The present study had a 12-week follow-up and, therefore, its extrapolation to the long-term effects should be done with caution. However, studies that tested the effects of treatments with SGLT2i on body composition reported that a large part of this effect occurs in the first weeks [1–4]. So, it is possible that in this study we have captured the main changes in body composition that occur with this medication and, hence, these findings may contribute to the broad spectrum of clinical benefits seen in clinical trials with SGLT2i.

This study has some limitations. It was not possible to measure changes in fluid volumes and, in this way, we cannot precisely indicate the components of the total mass that were affected by the treatment. This may be true even if a return to baseline levels of fluid volumes is expected after one-month of SGLT2i treatment [4]. According to the study protocol, no lifestyle intervention was recommended to study participants. For this reason and because it was a short-term follow-up, we did not evaluate changes in lifestyle. However, it is possible that small changes have occurred in both arms of the study and may at least partially influence the results

## Conclusions and implications

In summary, treatment for 12 weeks with dapagliflozin compared to glibenclamide increased the proportion of lean-to-total mass due to a more intensive reduction in fat mass than in lean mass. This finding suggests the use of SGLT2i may result in improvement of skeletal muscle metabolism even in individuals with reduced lean mass.
